# Inclusive and intersectoral: community health improvement planning opportunities to advance the social determinants of health and health equity

**DOI:** 10.1186/s12889-023-17496-5

**Published:** 2024-01-13

**Authors:** C. Ross Hatton, Rasika Kale, Keshia M. Pollack Porter, Yeeli Mui

**Affiliations:** 1 Department of Health Policy and Management, Johns Hopkins Bloomberg School of Public Health, 624 N Broadway, Baltimore, MD 21205 USA; 2 Department of International Health, Johns Hopkins Bloomberg School of Public Health, 615 N Wolfe St, Baltimore, MD 21205 USA

**Keywords:** Community health improvement plans, Social determinants of health, Health equity

## Abstract

**Background:**

Community health improvement plans (CHIPs) are strategic planning tools that help local communities identify and address their public health needs. Many local health departments have developed a CHIP, yet there is a lack of research on the extent to which these plans address root causes of health disparities such as the social determinants of health. This study aims to inventory the social determinants of health included in 13 CHIPs and examine facilitators and challenges faced by local health departments and partners when trying to include the social determinants of health.

**Methods:**

We conducted a comparative plan evaluation by scoring 13 CHIPs on their inclusion of equity orientation, inclusive planning processes, and five social determinants of health: health care access and quality, the neighborhood and built environment, economic stability, social and community context, and education access and quality. To supplement the plan evaluation, we conducted 32 in-depth interviews with CHIP leaders and stakeholders to understand the factors contributing to the inclusion and exclusion of the social determinants of health in the planning process.

**Results:**

CHIPs received an average score of 49/100 for the inclusion of the social determinants of health. Most plans addressed health care access and quality and the neighborhood and built environment, but they often did not address economic stability, the social and community context, and education access and quality. Regarding their overall equity orientation, CHIPs received an average score of 35/100, reflecting a relative lack of attention to equity and inclusive planning processes in the plans. Interviews revealed that challenges engaging partners, making clear connections between CHIPs and social determinants, and a lack of capacity or public and partner support often led to the exclusion of the social determinants of health. Recommendations to improve planning processes include improving data infrastructure, providing resources for dedicated planning staff and community engagement incentives, and centering equity throughout the planning process.

**Conclusions:**

Although local health departments can leverage CHIPs to improve population health and address health disparities, they face a range of challenges to including the social determinants of health in CHIPs. Additional resourcing and improved data are needed to facilitate broader inclusion of these determinants, and more work is needed to elevate equity throughout these planning processes.

**Supplementary Information:**

The online version contains supplementary material available at 10.1186/s12889-023-17496-5.

## Background

In recent decades, researchers, policymakers, and practitioners have increasingly recognized the need to address the social determinants of health (SDoH) to improve health equity and reduce disparities, many of which are rooted in long histories of structural racism and systemic exclusion from public resources, services, and programs [1–4]. Successful SDoH interventions in the U.S. have included moving families from high to low-poverty neighborhoods, supplementing the incomes of elderly adults, and providing early childhood education to children in families with low incomes, which can, among other outcomes, improve mental health, lower disability rates, and reduce medical costs, respectively [5]. However, efforts to address the SDoH face a host of challenges. For example, public health responses to so called “wicked” problems like the obesity epidemic have achieved isolated wins and little progress overall, despite efforts to intervene on individual level nutrition and physical activity since the 1990s [6, 7]. In addition, data and cost sharing, as well as institutional silos, have limited the formation of the partnerships and collaborations needed to address such complex public health issues [8, 9].

In line with a growing interest in whole systems approaches, a new paradigm of public health in the United States has called for intersectoral planning and coordinated action in which public health departments and leaders work with partners to strategize and ultimately address the SDoH [10, 11]. Public health leaders like the Centers for Disease Control and Prevention (CDC) have advocated for increased intersectoral planning to better integrate the SDoH in public health planning, policymaking, and practice [12]. Equipped to understand the distinctive needs of their communities, including vulnerable populations, local health departments (LHDs) are uniquely positioned to facilitate these efforts by informing local interventions, convening cross-sector partners, sharing and integrating data, and influencing how organizations and individuals in their communities behave [13, 14].

A pivotal activity that can assist in these efforts is community health improvement planning - a process that guides a public health department, its partners, and its stakeholders on the development of policies and accountability systems to improve population health within their jurisdiction. In its calls for continued work on the SDoH, the CDC has specifically identified these processes for their capacity to effectively mobilizing multisectoral partnerships [13]. Since 2011, the national voluntary accreditation program spearheaded by the Public Health Accreditation Board has required both a community health needs assessment (CHNA) and a CHIP as part of the documentation that health departments must submit for successful accreditation [15, 16]. Additionally, the revised requirements for tax-exempt status for nonprofit hospitals in the Patient Protection and Affordable Care Act of 2010 require a CHNA and the adoption of an implementation strategy, which often takes the form of a CHIP [17]. As of 2019, an estimated 71% of LHDs have participated in developing a CHIP. Most LHDs (63%) also collaborate with non-profit hospitals to develop CHNAs, and many non-profit hospitals also provide input on strategies to improve community health [18].

The CHNA, in which data are collected and integrated to identify areas of community health need and disparities, represents the beginning of the CHIP planning process. Leaders of the CHIP’s development then typically engage individuals and organizations in the community to prioritize specific community health needs, select strategic goals, and identify strategies to achieve these goals. To facilitate these efforts, many LHDs use tools like Healthy People 2020 (HP2020) and Mobilizing for Action through Planning and Partnerships (MAPP). HP2020 identifies public health goals and measurable objectives that health agencies can use when participating in or leading planning efforts to improve their communities’ health, while MAPP provides a planning framework by which local health system partners can convene to develop, implement, and evaluate CHIPs [19, 20]. Both tools emphasize the central role of addressing the SDoH to improve community health. HP2020 also emphasizes the need to address health equity, which it defines as “[eliminating] disparities and [improving] the health of all groups” [21]. Notably, the recently released MAPP 2.0 framework elevates and centers health equity relative to the original framework [22].

Despite the availability of tools like MAPP and the accompanying technical assistance provided by organizations like the National Association of City County Health Organizations (NACCHO), many LHDs experience constraints during the CHIP development process, and CHIPs often do not address the SDoH and health equity. For example, one study of LHDs in Florida found that, while many used the MAPP process, there were challenges with identifying and implementing strategic priorities as well as needing technical assistance to support these efforts [23]. LHDs and partners in Kansas have reported similar needs for training and technical assistance, particularly among rural counties, and an assessment of CHIPs in the Rocky Mountain Region and Western Plains identified resources, technical assistance, and maintaining partner commitment to the CHIP between plans as key challenges [24, 25]. In addition, a recent nationwide assessment of CHIPs from over 30 states found that plans generally contain few objectives related to addressing health disparities, particularly for racial, ethnic, and sexual minorities, suggesting that CHIPs can do more to address health disparities [26]. Similarly, an assessment of CHIPs in Illinois found that very few plans had priorities and interventions related to the SDoH [27].

Although some research has reviewed the inclusion of SDoH objectives in CHIPs, a more comprehensive assessment of the specific SDoH present and missing from CHIPs can reveal social determinants that need increased attention to alleviate health disparities [27, 28]. Furthermore, to our knowledge, there are unanswered questions regarding the challenges and facilitators of including the SDoH and health equity in CHIPs. These knowledge gaps are consequential for public health actions and leveraging CHIPs to address the SDoH and advance health equity. Understanding of the CHIP development process may provide valuable insight around best practices and opportunities for strengthening community health planning efforts. Therefore, the objectives of this study were to: (1) Inventory the SDoH domains included in CHIPs; (2) Examine the facilitators and challenges faced by LHDs and their partners when developing and adopting strategies that address the SDoH; and (3) identify opportunities to improve the capacity of LHDs and their partners to address the SDoH through the CHIP planning process.

## Study data and methods

This research involved two components: a descriptive content analysis of a sample of CHIPs and key informant interviews to understand perceptions of the CHIP development process.

### Sample plans

Using convenience sampling, this study focused on the most recent CHIP adopted at 12^1^ local health department demonstration sites, whose original plans were selected by NACCHO to serve as demonstration sites and examples of high-quality CHIPs. These plans received funding from NACCHO to support the planning process and ongoing technical assistance (e.g., identifying data needs, providing guidance on conducting focus groups, introducing CHIP leaders to different data sources and methods, etc.) [29, 30]. Having benefited from both financial and technical support, these plans were better equipped to manage some of the challenges that can arise during the planning process (e.g., inadequate resourcing and expertise). Sampled local health departments, more than half of which are accredited, covered different regions of the U.S. and served populations ranging from 20,000 to 1 million people across urban, suburban, rural, and frontier communities (Table 1).

**Table 1:** Summary of local health departments reviewed in the study sample

Agency /Consortium	Year of CHIP Adoption	State	Jurisdiction Level	Size of Population Served^a^	Urbanicity	Original Year of Accreditation
Alachua County Health Department	2020	Florida	County	278,468	Urban, Rural	2016
Austin/Travis County Health and Human Services Department	2018	Texas	City/County	1,290,188	Urban, Rural, Suburban	2016
Barry-Eaton District Health Department	2016	Michigan	County	109,175	Urban, Rural, Suburban	2016
Blue Ridge Health District^b^	2019	Virginia	Multi-County	212,567	Urban, Rural, Suburban	2015
Central Valley Health District	2018	North Dakota	Multi-County	23,469	Rural	2015
East Central Kansas Public Health Coalition	2012	Kansas	Multi-County^c^	103,152	Semi-urban, Rural, Frontier	Not Accredited
Gallatin City-County Department	2019	Montana	County	118,960	Rural, Suburban	2015
Ingham County Health Department	2019	Michigan	County	284,900	Urban, Rural, Suburban	2019
Kittitas County Health Department	2018	Washington	County	44,337	Rural	Not Accredited
New Orleans Health Department	2021	Louisiana	City	383,997	Urban	2014
Norwalk Health Department	2019	Connecticut	Multi-City	167,804	Urban, Suburban	2014
Plumas County Public Health Agency	2016	California	County	19,790	Rural, Frontier	2018
San Francisco Department of Public Health	2012	California	City/County	873,965	Urban	2017

^a^2020 US Census Bureau estimates

^b^The Thomas Jefferson Health District was renamed the Blue Ridge Health District on Jan. 1, 2021

^c^The East Central Public Health Coalition comprises Health Department representatives from 8 counties: Chase, Coffey, Franklin, Greenwood, Lyon, Morris, Osage, and Wabaunsee

### Plan evaluation instrument design

Plan evaluation scholars have studied the quality of plans, since the early 1990s, to identify their strengths and weaknesses and judge their overall quality based on standardized criteria. In using these standards of good practice, plan evaluations function as a learning tool to improve the content, quality, and processes of existing and future plans [31–36]. While historically this body of work has covered diverse topics, including sustainable development, affordable housing, natural hazards, and environmental protection [37], plan evaluations have recently directed more explicit attention to public health [38], including community design [39, 40], physical activity [41], and food systems [36, 42].

A plan evaluation protocol was developed in line with standard methods of prior plan evaluation studies described above. First, a new evaluation instrument (Additional file 1: Appendix A) was developed, comprising a framework to assess the extent to which CHIPs address the SDoH. Healthy People 2030 (HP2030) was used to define five SDoH domains: 1) economic stability, 2) education access and quality, 3) health care access and quality, 4) neighborhood and built environment, and 5) social and community context. To avoid double coding, we retained objectives related to housing under the neighborhood and built environment domain and objectives related to promoting positive relationships at home, at work, and in the community (e.g., health literacy, family and community relationships, and bullying) under the social and community context domain. Because efforts to address food access and healthy eating are often cross-cutting, involving multiple domains, we coded objectives related to food security, access, and healthy eating under the domain they most closely aligned like the neighborhood and built environment domain (e.g., access to healthy food retail).

Second, in order to assess the quality of plans, standard content analysis techniques were applied whereby for each SDoH domain, we evaluated on plan quality characteristics related to fundamental elements of a plan: goals and objectives (i.e., statements of future desired conditions that drive the plan and proposed actions), data (i.e., analysis of baseline and/or future conditions), proposed strategies (i.e., actions or recommendations to achieve the stated goals), and monitoring and evaluation (i.e., indicators to track progress toward stated goals). To evaluate plan quality characteristics related to equity, we drew from a plan equity evaluation tool developed by Loh and Kim, who broadly define equity in planning as increasing agency and expanding access to resources and opportunities for those who are marginalized. Importantly, a plan oriented toward equity would also be created through an inclusive public participation process [43]. Therefore, additional indicators were included to assess how equity appeared in plans, whether underserved communities were identified, and whether obstacles to implementing equitable policies were discussed. Additionally, we evaluated characteristics of the planning process, including descriptions of the public participation process, how officials engaged historically marginalized groups, and how community feedback was incorporated. Finally, given growing concerns around coordination in local planning, one indicator was included to determine the level of integration of proposed strategies across the SDoH domains [44].

A total of 28 indicators were used to evaluate plans. Following other plan quality evaluations, each indicator in the evaluation instrument was assigned a score on of 0 (not present), 1 (present, but limited in scope or detail), or 2 (present with details, comprehensive, and actionable). Plans could receive a maximum total score of 40 for the five domains of the SDoH (i.e., a maximum score of 2 for each fundamental element of a plan: goals and objectives, data, proposed strategies, monitoring and evaluation); 2 for integration of proposed strategies, and 14 for overall equity orientation including inclusive planning processes (i.e., a maximum score of 2 for each equity indicator).

### Sample of key informant interviews

Interviews were first conducted with personnel identified in CHIP planning documents as leaders of the development process. Using snowball sampling, we recruited additional key informants who were identified as other key participants (e.g., staff at partnering hospitals, non-profits, and public agencies) involved in the development of their jurisdiction’s CHIP. We attempted to contact CHIP personnel a minimum of three times before ending their recruitment. Interviews took place over Zoom between March and September 2022.

To better understand why certain SDoH were or were not included in these CHIPs and what opportunities there might be for advancing their integration in CHIPs, we constructed the interview guide to align with how each plan scored on the SDoH evaluation instrument. Specifically, we began each interview by asking participants to describe the CHIP planning process, their role and the role of their organization in the CHIP’s development, and the goals and aspirations of their community’s CHIP. We then asked participants to describe how community partners were involved in the plan’s creation and how CHNA data guided the CHIP’s goals and priorities. Finally, we asked a core set of questions about CHIP strategies related to the SDoH domains (e.g., economic stability). In cases where a plan did contain actions related to a given domain, we asked what the actions were trying to achieve, who was involved in their creation, whether and how proposed actions would reduce inequities, and what, if any, facilitators or challenges there were to including these actions. For plans that did not include actions related to a given domain, we asked whether these actions were discussed and whether there were any reasons for their exclusion from the CHIP. The research team met regularly over the course of conducting interviews and iterated on the guide as key informant perspectives materialized. Each interview was conducted via Zoom, audio-recorded with the informant’s permission, and ranged from 60 to 90 min.

### Data analysis

We analyzed the extent to which CHIPs addressed the SDoH by calculating individual and overall average scaled scores for the five SDoH domains, integration of proposed strategies, and equity orientation including inclusive planning processes. Consistent with prior plan evaluation approaches, CHIP scores were summed and then divided by the total number of maximum points for both the full plan and for each SDoH domain [42]. These scores were then multiplied by 100. Rescaling of scores from 0 to 100 facilitated comparisons across individual SDoH domains. We also compared the raw average scores for each fundamental element of plans (i.e., data, strategies, goals, and monitoring) by SDoH domain to help explain variation in the respective SDoH domain scores.

To supplement the descriptive content analysis, transcripts from key informant interviews were analyzed using Nvivo, Version 1.0 [45]. We first constructed a codebook using deductive codes based on our research questions and interview guide. In addition, we used memos and research team meeting notes written during the process of conducting interviews to identify additional inductive codes. Two coders piloted this initial codebook on 7 of the 32 transcripts and discussed coding discrepancies with the research team until reaching consensus. Both during and after pilot coding, the team continued to meet to modify, remove, and add codes as necessary. The final codebook was used by two coders to independently code each half of the 32 transcripts. We examined similarities and differences between codes to identify emergent thematic patterns across interview transcripts. Illustrative quotes were provided to add context and clarity on themes.

## Results

### How CHIPs address the SDoH

**Fig. 1:**
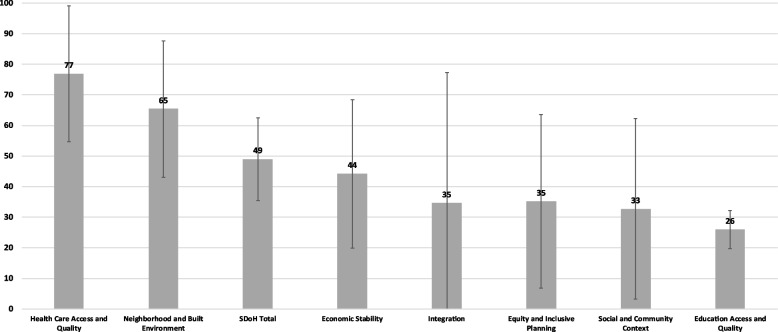
Average scaled scores for the SDoH domains, integration, and overall equity orientation Authors’ Note: Plan scores were summed and then standardized for both the aggregate SDoH score and each individual SDoH domain. Scores were standardized by dividing scores by the maximum possible score (e.g., scores were divided by 8 for each of the SDoH domains). The bars represent the average of these standardized scores across the 13 plans, while the error bars represent the standard deviation

Figure 1 presents individual and overall average scaled scores across the five SDoH domains, integration or proposed strategies, and equity orientation including inclusive planning processes. Additional file 1: Appendix B identifies individual plan scores. Overall, CHIPs received an average SDoH score of 49 out of 100 and ranged from 30 to 70 (not shown). In terms of the SDoH included in CHIPs, the health care access and quality domain received the highest score. Twelve of the 13 plans received a score of 63 and above in the health care access and quality domain, with an average score of 77. CHIPs often either centered general health care access as a strategic priority or elevated access to specific health care services as a central goal of the plan. For example, one community prioritized access to behavioral health care services as one of its primary goals [46].

CHIPs also often addressed the neighborhood and built environment domain, though to a lesser extent than health care access and quality. For the neighborhood and built environment domain, CHIPs received an average score of 65, with scores ranging from 25 to 100. Although built environment goals were not typically among plans’ strategic priorities, they were frequently included as part of a strategy to address other health priorities. For example, efforts to address chronic disease, obesity, nutrition, and access to care were often accompanied by strategies to improve the built environment. In Greater Norwalk, for example, pedestrian infrastructure and active transport opportunities were recommended to address chronic disease and obesity [47].

Economic stability, the social and community context, and education access and quality were comparatively less prioritized in CHIPs. The economic stability domain received an average score of 44, ranging from 25 to 88, and the social and community context domain received an average score of 33, ranging from 0 to 88. The Blue Ridge Health District (BRHD) CHIP was the only plan to include clearly defined actions in both domains. Relative to the other SDoH domains, education access and quality averaged the lowest score of 26, ranging from 13 to 38. This reflected the fact that education was often the least prioritized domain in each of the 13 plans.

To examine potential drivers of the overall differences between CHIPs addressing each SDOH, we also compared raw scores for each fundamental element of plans across the five SDoH (Table 2). CHIPs received relatively higher scores in relation to data informing all five domains, with an average score of 1.7. In other words, CHIPs often presented many types of data (e.g., quantitative, qualitative, and spatial) on a broad range of health and SDoH topics (e.g., chronic disease, food insecurity, poverty, etc.). In contrast, CHIPs received scores below 1 in relation to proposed strategies, goals and objectives, and monitoring and evaluation – meaning these fundamental elements of plans were often present but limited in scope or detail – though we found variability across the SDoH domains. CHIPs tended to score relatively lower on these fundamental elements of plans when addressing economic stability, social and community context, and education access and quality, compared to health care access and quality and the neighborhood and built environment. Notably, CHIPs scored particularly poorly on proposed strategies, goals and objectives, and monitoring and evaluation when it came to education access and quality. Most plans lacked strategies and goals related to this domain, and no plans included clear measures or plans to evaluate proposed strategies related to education.

**Table 2:** Average scores for each fundamental element of plans by SDoH domain

Domain	Data	Proposed Strategies	Goals & Objectives	Monitoring & Evaluation
Health Care Access and Quality	1.9	1.6	1.6	1.1
Neighborhood and Built Environment	1.8	1.4	1.2	0.8
Economic Stability	1.9	0.8	0.5	0.3
Social and Community Context	1.2	0.5	0.5	0.4
Education Access and Quality	1.8	0.2	0.1	0.0
**Average**	**1.7**	**0.9**	**0.8**	**0.5**

Authors’ Note: Plans could receive a 0, 1, or 2 for each fundamental element of a plan. The above scores reflect the averages of these scores across all 13 plans

With regard to integration, only three CHIPs recommended actions that could support integrated strategies across the SDoH domains. Notably, a central cross-cutting theme of the 2018 Austin and Travis County CHIP was transportation, which the plan elevated as means to alleviating several SDoH [48]. However, most plans (*n* = 7) did not recommend actions across all five domains or actions that could clearly support integrated change and, therefore, received a 0, resulting in an overall average score of 35 (out of 100) for integration. Similarly, CHIPs received an average score of 35 for overall equity orientation and inclusive planning processes, reflecting generally limited discussion related to equity as well as vague descriptions of the public planning processes used to both inform and develop the CHIPs. Of the 13 plans, only four CHIPs received scores above 50 for their equity orientation and inclusive planning processes.

### Experiences with including the SDoH in CHIPs

Using snowball sampling, we spoke with 32 key informants involved in the development of 11 of the 13 CHIPs (one declined to participate and one could not be contacted). The number of interviews with staff from each jurisdiction ranged from one to five. In total, we spoke to 15 staff from LHDs, five staff from partnering hospitals, four staff from other local agencies (e.g., department of transportation), two staff from partnering universities, and six staff from other community partners (e.g., non-profit organizations).

Table 3 presents themes, sub-themes, and illustrative quotes related to facilitators and challenges of including the SDoH in CHIPs. Themes related to facilitators included the role of health disparities, local buy-in and interest in the CHIP, and public and partner support. Themes related to challenges included public and partner support, perception of community need, connections between the SDoH and CHIP, and system capacity and integration. In the following section, we describe these themes in greater detail and, where applicable, note how they contributed to observed differences in the extent to which each of the five domains were included in CHIPs.

**Table 3:**
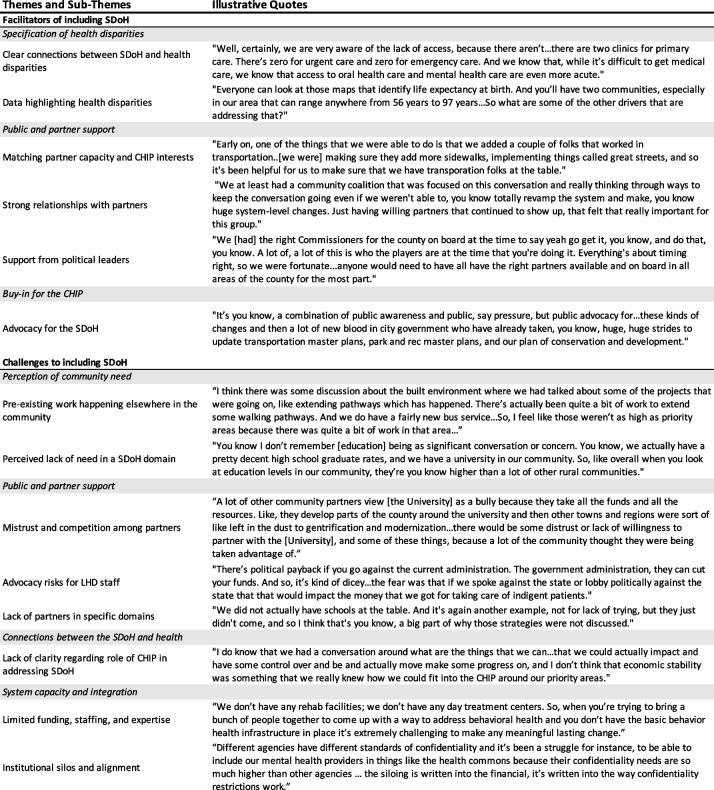
Facilitators and challenges to including SDoH in CHIPs: key themes and illustrative quotes

### Facilitators of including the SDoH

Three key themes emerged related to facilitators of including the SDoH in CHIPs: specification of health disparities, public and partner support, and buy-in for the CHIP. Within these themes, six sub-themes emerged, which we discuss in greater detail in the following section.

#### Specification of health disparities

By far the most common reason SDoH-related actions were included in CHIPs was because they addressed specific disparities, and CHIP leaders and partners could clearly articulate these connections. Key informants often discussed how their planning partners increasingly recognized that the disparities experienced by populations stemmed from wider structural inequalities. Multiple individuals described a clear understanding of how health care access and quality and the neighborhood and built environment impacted health, particularly through geographic access to services and resources. For example, one community partner noted that their neighborhood had few primary clinics and zero nearby urgent or emergency care facilities, highlighting the need to address barriers related to transportation and access.

“Well, certainly, we are very aware of the lack of access, because there aren’t…there are two clinics for primary care. There’s zero for urgent care and zero for emergency care. And we know that, while it’s difficult to get medical care, we know that access to oral health care and mental health care are even more acute.” – University Partner

These visible disparities underscored the impact of limited access to services and resources on health outcomes, while data from the CHNA further affirmed the connections between the SDoH and public health problems. Indeed, CHIP leaders and partners often identified priority communities through population data analysis during the development of the CHNA or through advocacy by CHIP partners whose experiences working in underserved communities helped inform opportunities to improve health through addressing the SDoH. For example, health systems data identifying uninsured populations or longitudinal metrics identifying high-needs neighborhoods were utilized by several jurisdictions in the CHIP development process:

“Everyone can look at those maps that identify life expectancy at birth. And you’ll have two communities, especially in our area that can range anywhere from 56 to 97 years…So what are some of the other drivers that are addressing that?” – Hospital Partner

#### Public and partner support

Key informants also highlighted the critical role of public and partner support for advancing actions related to the SDoH. In many cases, actions related to the SDoH developed because partners in the planning process realized they had aligned interests and untapped capacity. For instance, in one community, this type of collaboration supported efforts to improve access to sidewalks and active transport by tapping into the capacity of transportation partners:

“Early on, one of the things that we were able to do is that we added a couple of folks that worked in transportation..[we were] making sure they add more sidewalks, implementing things called great streets, and so it's been helpful for us to make sure that we have transportation folks at the table.” – Health Department Staff

Although these partnerships were broadly beneficial, many key informants noted the role that partnerships played in fostering the inclusion of actions specifically related to heath care access and quality and the built and neighborhood environment. For example, key informants described how strong partnerships with those who had funding or expertise (e.g., in improving access to health services, transportation) helped bring those resources into the planning process. In some communities, partner coalitions external to the CHIP planning process (e.g., coalitions around oral health, social safety net programs, behavioral health, etc.) aided in building an extended network that could be tapped into during the CHIP’s development. One key informant noted that making changes to their local behavioral health system was a large task that required sustained interest from many partners in order to be successful. Strong relationships and coalitions were ultimately key to sustaining this participation, even if progress was incremental or slow:

“We at least had a community coalition that was focused on this conversation and really thinking through ways to keep the conversation going even if we weren’t able to, you know totally revamp the system and make, you know huge system-level changes. Just having willing partners that continued to show up, that felt that really important for this group.” – Health Department Staff

In addition to strong partners, key informants noted the importance of local political support, particularly for addressing the built and neighborhood environment, as actions to address this SDoH domain often required substantial investments in land and infrastructure. For example, adding sidewalks to improve walkability in one community was highly political because it required taking a portion of residents’ land. A lengthy engagement with community members helped to build the support necessary to plan for and ultimately advance an expansion of sidewalks. In another community, the mayor was a major proponent of active transportation, which ultimately led to the development of a task force and commission that elevated the need to expand opportunities for walking and biking, which were then included in the respective CHIP.

#### Buy-in for the CHIP

Key informants also identified the importance of local buy-in and interest in the CHIP from partners, policymakers, and the broader community as key facilitators. More specifically, individuals noted that community awareness and advocacy for the SDoH helped facilitate the inclusion of related actions in CHIPs in three ways. First, recognition that the CHIP was an appropriate vehicle to address the SDoH led to greater support among these committed partners for including related goals and strategies. Second, this buy-in from community partners helped foster the collaborations needed to design and implement proposed strategies. Finally, elected officials in communities with broader buy-in and interest offered funding and opportunities to integrate the CHIP with other planning processes:

“It’s you know, a combination of public awareness and public, say pressure, but public advocacy for…these kinds of changes and then a lot of new blood in city government who have already taken, you know, huge, huge strides to update transportation master plans, park and rec master plans, and our plan of conservation and development.” – Health Department Staff

### Challenges to including the SDoH

Four themes emerged related to the challenges key informants experienced when trying to include the SDoH in their CHIPs: (1) perception of community need, (2) public and partner support, (3) connections between the SDoH and health, and (4) system capacity and integration. Among these themes, we identified eight sub-themes, which we describe in this section.

#### Perception of community need

Much of the work included in CHIPs reflected a recognition that there was a disparity in the community that needed to be addressed. A perception of community need, often informed by data or testimony from partners, enabled the inclusion of many strategies. Yet, in some cases, CHIP leaders perceived a lack of need related to the specific SDoH, which led to their exclusion. This was the case for several key informants with regard to the neighborhood and built environment. Many individuals believed that partners were already leading efforts in this domain, which further de-emphasized the perceived need to address it through the CHIP process:

“I think there was some discussion about the built environment where we had talked about some of the projects that were going on, like extending pathways which has happened. There’s actually been quite a bit of work to extend some walking pathways. And we do have a fairly new bus service…So, I feel like those weren’t as high as priority areas because there was quite a bit of work in that area…” – Hospital Partner

Key informants reported similar reasons when discussing why education was excluded, though, whereas they often perceived prior work among partners as contributing to a lack of community need in the built environment, the perceived lack of need in the education access and quality domain appeared to be the result of how key informants engaged with education-related data. Multiple key informants described education at the population level and rarely mentioned disparities in access or quality:

“You know I don’t remember that being as significant conversation or concern. You know, we actually have a pretty decent high school graduate rates, and we have a university in our community. So, like overall when you look at education levels in our community, they’re you know higher than a lot of other rural communities.” – Health Department Staff

#### Public and partner support

Although public and partner support was often identified as a facilitator of including the SDoH, it was also an obstacle in many jurisdictions. Both mistrust and competition among partners challenged leaders’ efforts to identify and include certain strategies. In some cases, partners at the table had conflicting internal priorities that narrowed the strategies and actions they supported. For example, one CHIP sought to improve access to fruits and vegetables in convenience stores, but selling fruits and vegetables at affordable costs was out of sync with store owners’ profit goals. In other cases, mistrust limited partners’ willingness to work together to help underserved populations. In one jurisdiction, larger organizations such as the local university were perceived as extracting resources and investing only in its own interests, leaving other parts of the community behind. These pre-existing tensions impacted the degree of mutual trust and partners’ interest in collaborating:

“A lot of other community partners view [the University] as a bully because they take all the funds and all the resources. Like, they develop parts of the county around the university and then other towns and regions were sort of like left in the dust to gentrification and modernization…there would be some distrust or lack of willingness to partner with the [University], and some of these things, because a lot of the community thought they were being taken advantage of.” – Health Department Staff

As leaders of the CHIP, LHD staff also often struggled to walk the line of advocacy. Many strategies intended to address the SDoH require substantial investment and systems change in communities, but LHD staff faced the possibility of reprisal if they were seen as sponsoring specific policies in conflict with state leadership. In one community, staff feared that disagreeing with the state government or advocating for specific policies risked undoing efforts to address health disparities, as the state could reduce funding for indigent patients:

“There’s political payback if you go against the current administration. The government administration, they can cut your funds. And so, it’s kind of dicey…the fear was that if we spoke against the state or lobby politically against the state that that would impact the money that we got for taking care of indigent patients.” – Health Department Staff

Finally, in some cases, there were no partners at the table to advocate for or advance strategies related to specific domains. Because many strategies were included as a result of specific partners’ advocacy or partnerships that emerged during the planning process, the absence of key partners could effectively eliminate the possibility of addressing certain SDoH. For example, key informants often noted that school systems and educational partners were difficult to engage in the planning process. Without the involvement of educational partners, strategies to address this SDoH domain were almost always left out.

#### Connections between the SDoH and health

For some domains (e.g., health care access and quality, the built and neighborhood environment), the connection to health, as well as the role of the CHIP in addressing this connection, was evident. For other SDoH, like economic stability, several key informants felt that there was an unclear connection. A lack of direction for how proposing actions to support steady incomes fit within the purview of CHIPs or uncertainty about whether CHIP partners had the skills and relationships needed to implement the desired changes led to their exclusion from plans:

“I do know that we had a conversation around what are the things that we can…that we could actually impact and have some control over and be and actually move make some progress on, and I don’t think that economic stability was something that we really knew how we could fit into the CHIP around our priority areas.” – Healthcare Partner

#### System capacity and integration

One of the chief challenges to including the SDoH in CHIPs was limited capacity. Leaders of the development process frequently shared that limited funds, staffing, expertise, and infrastructure (e.g., lack of food retail, lack of substance use treatment facilities, etc.) limited the types of actions considered or ultimately adopted, particularly when it came to addressing issues related to economic stability and the social and community context. For rural communities in particular, these challenges limited the scope of the CHIP. For example, in one rural community the lack of behavioral health care providers restricted what CHIP partners could ultimately include in the plan:

“We don’t have any rehab facilities; we don’t have any day treatment centers. So, when you’re trying to bring a bunch of people together to come up with a way to address behavioral health and you don’t have the basic behavior health infrastructure in place it’s extremely challenging to make any meaningful lasting change.” – Hospital Partner

Finally, key informants also identified fragmentation of local networks and institutional silos as additional challenges throughout the planning process. For example, disconnected networks made it difficult to map existing work among partners. Some communities, for instance, had a wealth of partners and working committees within their jurisdictions but did not have a consolidated resource to identify all relevant partners and activities that could be leveraged in the CHIP*.* Partners also often had different data and confidentiality standards, making data sharing that could help prioritize actions to address health disparities more challenging. For example, one key informant identified siloed healthcare data and confidentiality issues as a barrier to integrating service planning and delivery:

“Different agencies have different standards of confidentiality and it’s been a struggle for instance, to be able to include our mental health providers in things like the health commons because their confidentiality needs are so much higher than other agencies … the siloing is written into the financial, it’s written into the way confidentiality restrictions work.” – Non-profit Organization Partner

### Recommendations from CHIP leaders and partners

Key informants also reflected on their needs and recommendations for how to improve the CHIP planning process and foster greater inclusion of the SDoH. Table 4 presents recommendations that emerged around three key themes: (1) center equity and structural change, (2) improve collaboration, break down barriers, and (3) revise logistics, planning infrastructure. Table 4 presents these themes alongside specific examples provided by key informants.

**Table 4:**
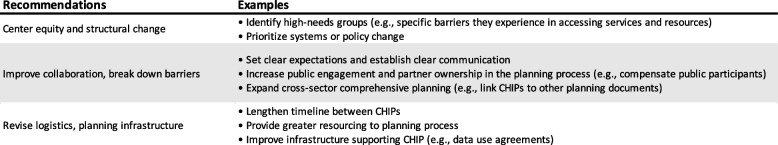
Recommendations to Improve CHIP Planning Process and Foster Inclusion of the SDoH

#### Center equity and structural change

Some key informants believed their planning processes could have more fully addressed the SDoH by further centering health disparities and emphasizing strategies related to structural (e.g., policy) change. For example, one LHD staff member noted that their community’s planning process did not prioritize identifying and mitigating heightened barriers to accessing services and resources among vulnerable subpopulations, and as a consequence, the CHIP reflected this gap in its relative lack of attention to health disparities. In another community, a hospital partner whose CHIP scored highly noted that, while proud of the CHIP, much of the planning process focused on individual-level strategies (e.g., behavior change) as opposed to upstream policy changes. Because of this, their community had decided that future planning would more intentionally center policy change in its CHNA and CHIP, with the goal of producing actions that could make more substantive impacts on the SDoH.

#### Improve collaboration, break down barriers

Reflecting a recognition that community partner participation often facilitated and shaped the SDoH actions included in CHIPs, key informants often spoke of the need for improved collaboration. Some noted that more clearly communicating expectations for participation in the planning process could help ensure time and resource commitment from partners throughout the CHIP’s development. For example, key informants identified memorandums of understanding (MOUs) as a tool that could improve communication, expectation setting, and accountability. Many also indicated that CHIP leaders needed to do a better job engaging partners and the public in the planning process. For example, one key informant noted that, without these voices, CHIP priorities and actions might fail to align with actual community needs. Key informants mentioned providing incentives like childcare and financial compensation as strategies that might help enhance community engagement, as well as diversifying methods of qualitative data collection and community input (e.g., focus groups, surveys, photovoice, etc.). Another noted that giving partners more ownership of the process could help make the CHIP more holistic in its approach to improving community health. Finally, some key informants indicated that local strategic plans could link to each other (e.g., comprehensive plans) to improve partner and resource alignment and help overcome challenges related to data sharing between institutions.

#### Revise logistics, planning infrastructure

Many key informants also expressed a need to revise the infrastructure and logistics of the CHIP planning process. Several noted that the requirement that CHNAs be completed every 3 years lead to short timelines between CHIPs, which can rush the planning process and make it difficult to observe any progress in indicators between plans. Some suggested that lowering the frequency (e.g., every 5 years) would help communities develop more robust plans and promote more meaningful progress in their priorities.

Altogether, the most significant needs CHIP leaders and partners identified were related to resources and improving the infrastructure supporting the development of CHIP. Greater dedicated funding could help LHDs more effectively staff the planning process and compensate community members to improve community engagement. Similarly, some key informants noted the need for improved planning infrastructure. Many people involved in the development of CHIPs had full-time jobs, making the CHIP secondary to those responsibilities. As an alternative to relying on people with other commitments to run the process, key informants suggested the use of regional coordinators and dedicated ‘backbone staff’ to facilitate engagement and planning efforts, though the use of these external facilitators would again require greater investment in the CHIP planning process. Lastly, the use of MOUs and data use agreements was suggested to ensure fair and transparent data access during the development of the CHNA and CHIP.

## Discussion

This study represents a first look at how CHIPs address the SDoH, presenting the results of a content analysis coupled with qualitative findings from key informants who led the development of CHIPs in different jurisdictions across the country. Results from this analysis extend the existing literature on community health planning with important implications for efforts to further the success of CHIPs to address the SDoH and advance health equity.

Interviews with LHD staff and partners highlighted how their understanding of the connections between the SDoH and health disparities, as well as the availability of data informing these connections, facilitated the inclusion of SDoH in CHIPs. Given previous research has found that CHIP priorities often do not address health disparities [26], findings from this study suggest potential shortcomings in the availability and types of data used for the CHNA and CHIP. Indeed, a study of 10 model CHIPs found that the data used for CHNAs and CHIPs was typically at the county level and could not be used to identify sub-populations or disparities of interest (e.g., health disparities by socioeconomic status) [49]. In addition, our findings indicate that CHIP participants may more readily understand the connections between certain SDoH (e.g., the built and neighborhood environment) and health than other SDoH domains (e.g., economic stability). Therefore, future research should further investigate how CHIPs can better engage in these less traditional public health topics, such as economic stability and development, by drawing clearer connections with health and health disparities, including bolstering the measurement of them, especially at the local level.

Findings also revealed nuances about the ways in which partnership dynamics affected the CHIP development process. Strong collaboration and relationship-building between partners and with the broader community was reported to be one of the greatest facilitators of CHIP development, which aligns with existing literature on public health management and governance that emphasizes the importance of trust and relationship building [25, 50–52]. We learned that mistrust of even a single partner (e.g., a local university) can impede efforts to collaboratively address the SDoH, despite overall high levels of trust between partners [50]. Additionally, diversity in partner engagement can foster the inclusion of SDoH in CHIPs, particularly in smaller jurisdictions [53].

Even when CHIP partner interests aligned in addressing the SDoH, limited organizational and system capacity undermined related efforts. These results corroborate existing literature documenting capacity challenges in community health planning and programming, especially around the need for technical expertise [54, 55]. Staffing, resources, and expertise, when lacking, often precluded efforts to address the SDoH through the CHIP. In one study, Carroll et a. identified jurisdictional size as a factor influencing inclusion of the SDoH in CHIP development and suggested that health departments covering larger jurisdictions may have more resources and technical expertise facilitating this [53]. This distinction was less clear in our data, as jurisdictions described organizational capacity as a challenge for CHIP development regardless of size. However, health departments with larger partner networks and resource bases (e.g., Alachua, Austin, Blue Ridge) also scored higher in our evaluation for the SDoH inclusion [48, 56, 57]. While funding was understood to be a universal challenge, health departments may have differing specific capacity building needs based on jurisdictional size that future work should aim to clarify.

Recommendations from key informants to improve the development process and foster greater inclusion of the SDoH focused on centering equity and structural change, improving collaboration and breaking down barriers, and revising CHIP logistics and infrastructure. Calls for centering equity and structural change aligned with our finding that plans scored poorly on overall equity orientation and language around inclusive planning processes. Several studies have described how equity in planning, broadly, is consequential for the distribution of resources and opportunities towards systematically disadvantaged and marginalized populations [43, 58], and equity in community health planning is no exception. One way to build capacity for centering health equity in CHIPs is by encouraging local health departments and CHIP developers to complete an organizational health equity capacity assessment upfront, to help them assess their understanding of and readiness for health equity [59]. This exercise could not only promote a greater understanding of equity but also use of a health equity lens when completing the CHIP. In addition, NACCHO recently updated its MAPP tool to elevate health equity and disparities in the planning process. Centering equity in tools like MAPP, which are used by many communities to develop their plans, may help to foster greater inclusion of the SDoH and health equity in CHIPs moving forward. Future work should prioritize examining how revisions to the MAPP framework impact the development and implementation of CHIPs.

Community partners played a key role in shaping CHIP strategies related to the SDoH, but many key informants nonetheless expressed a need for both improved partnership and greater community engagement, which is consistent with past literature identifying a lack of commitment from community partners as a major challenge for CHIPs [25]. Although LHDs frequently lead the development of CHIPs, the plans are intended to be multi-sectoral and collaborative, and these partnerships can help empower communities and their LHDs to address SDoH alongside issues more traditionally viewed as within the purview of LHDs (e.g., sexually transmitted infections). Ultimately, residents, stakeholders, and experts should be able to come together to engage in shared plan and decision making where at least some power is transferred to nonexperts [43, 60–62]. In other collaborative decision-making processes, group model-building or World Café-style discussions have been utilized to establish a common understanding of broader community needs and the value of addressing the SDoH through the CHIP, which may help to improve community member participation—a shared goal of many of the jurisdictions that participated in this study [63, 64]. Pre-existing local coalitions (e.g., social safety net coalitions) also helped strengthen relationships and support broader partner participation in the planning process; therefore, establishing and maintaining similar arrangements can help improve collaboration between local partners during a CHIP’s development.

But even for jurisdictions with both strong partnerships and a clear understanding of the connections between the SDoH and health disparities, limited infrastructure and resources available to support CHIP planning processes impeded efforts to address the SDoH through the CHIP. For example, many jurisdictions experienced challenges with data sharing, lacked technical expertise, and expressed a need for improved data infrastructure. Data gaps in general are hindering learning and progress towards equitable health outcomes [65–67]. Example MOUs, as well as dedicated legal staff and resources, is a potential strategy to help ensure that effective data sharing agreements are in place throughout the planning process [68]. Additionally, existing innovations in the field that could address these challenges include data sharing initiatives like the Data Across Sectors for Health (DASH) initiative funded by the Robert Wood Johnson Foundation [69], that provide a single streamlined platform for partners to share and view public health data. Policy and regulatory changes that allow for aggregate health data sharing could also enhance collaboration. For instance, Rosenbaum (2016) identified potential modifications to IRS regulations that would strengthen the ability of non-profit hospitals to implement CHNAs [70].

More broadly, the inclusion of the SDoH in CHIPs requires dedicated staff and additional funding to help facilitate strategic partnerships and planning. For rural jurisdictions in particular, health department staff were often stretched thin and had little capacity to lead the planning process and monitor progress. Having a dedicated “taskmaster” would help to keep planning processes on track, improve partner engagement, and promote accountability. Still, even larger and more well-resourced jurisdictions described the need for “backbone support” to support engagement and the process of prioritizing, executing, and monitoring the CHIP, as many partners at the table have jobs beyond their participation in the CHIP. Indeed, resource limitations are often described as one of the biggest challenges to CHIP planning efforts [25, 52]. Other key informants similarly noted that having someone lead who doesn’t have a “vested interest” in any specific public health issue would help manage the diverse voices and interests at the table. Finally, resources like childcare and stipends were also identified as crucial to improving public participation. Without these supports, public engagement, which key informants identified as important for ensuring CHIP priorities align with community needs, may continue to flounder. In sum, greater investment in CHIP planning infrastructure can potentially help sustain planning efforts, manage diverse partner interests, and improve partner and community engagement.

There are various limitations of this study. First, the 13 plans evaluated may not be representative of all CHIPs. However, their first iterations were considered model plans due to their receipt of support from NACCHO, and therefore, served as a starting place to explore our research questions. Second, key informants from two of the 13 plans did not participate in the interviews, and it is possible that their insights would have differed from other interview participants. However, leaders from one of these plans, Barry-Eaton, worked closely with Ingham County leaders during their planning process, and Eaton County is adjacent to Ingham County, suggesting that their insights and experiences might be similar. Third, the dates of each jurisdiction’s most recent CHIP varied, and it is possible that recall bias differentially impacted what the key informants recalled about their plans’ development. To help address recall bias, we shared the plans before the interviews and encouraged key informants to read and reference the plans directly during the interviews. Finally, state governments may vary in both the support they provide and the requirements they set for local CHIPs. This study does not explicitly capture this variation, which may further explain differences between plans, but key informants were free to share their experiences of state funding and requirements during interviews.

Despite these limitations, this study extends the literature on the inclusion of the SDoH in CHIPs and reveals opportunities to further understand how CHIP participants strategically plan to address population health and health disparities in their communities. First, to the extent that CHIPs address the SDoH, additional research is needed to understand how successful these efforts are in achieving their desired outcomes. Second, greater attention on engaging marginalized or historically disadvantaged is warranted. Specifically, researchers should examine best practices for engaging marginalized populations, as well how improved engagement translates to CHIP development and implementation. Third, given the importance of partner engagement and trust, future work should examine how these relationships and organizational structures (e.g., local coalitions) impact CHIP development. Finally, research is needed to understand whether and how incorporating equity in the planning process (e.g., through the MAPP framework) leads plans to more directly addressing health equity and disparities.

## Conclusion

CHIPs have the potential to improve population health, yet no study has evaluated how they address different domains of the SDoH. This study is the first to examine the extent to which some CHIPs address the SDoH and describe the factors that shape their inclusion and exclusion. Our findings suggest that there are notable gaps in the inclusion of economic stability, the social and community context, and education access and quality in these plans. However, there are also opportunities to further their inclusion by improving data infrastructures, expanding CHIP resources, broadening community engagement, and centering equity.

### Supplementary Information

**Additional file 1: Appendix A.** Sample instrument. **Appendix B.** Individual plan scores.

## Data Availability

The datasets generated and/or analyzed during this study are not publicly available due to participant confidentiality but are available from the corresponding author on reasonable request.

## Abbreviations

BRHD: Blue Ridge Health District
CHIP: Community health improvement plan
SDoH: Social determinants of health

## Footnotes

1 Barry-Eaton and Ingham County were originally part of a single jurisdiction alongside Clinton County: the Healthy! Capital Counties. Since the original joint plan, Barry-Eaton and Ingham have pursued separate CHIPs, and we evaluated those plans separately, resulting in a total of 13 CHIPs.
