# Supplementary material for: Inclusive and intersectoral: community health improvement planning opportunities to advance the social determinants of health and health equity
Source: BMC Public Health. 2024 Jan 13;24:170. doi: 10.1186/s12889-023-17496-5 (PMC10790276; doi:10.1186/s12889-023-17496-5)
Supplement: Supplementary file 1 — Additional file 1: Appendix A. Sample instrument. Appendix B. Individual plan scores. [file 12889_2023_17496_MOESM1_ESM.docx]

**Appendix**

**Appendix A. Sample Instrument**

**Appendix B. Individual Plan Scores**
